# The microbial strategies for the management of chemical pesticides: A comprehensive review

**DOI:** 10.1016/j.crmicr.2025.100519

**Published:** 2025-11-23

**Authors:** Ajay Kumar, Manoj Kumar Solanki, Manish Kumar, Amit Kaushik, Aditi Arya, Mahaswetta Saikia, Vivek Kumar Gaur, Rahul Prashad Singh, Sandeep Kumar Singh, Vipin Kumar Singh, Laurent Dufossé

**Affiliations:** a Amity Institute of Biotechnology, Amity University, Sector-125, Noida, Uttar Pradesh 201313, India; b Department of Life Sciences and Biological Sciences, IES University, Bhopal, Madhya Pradesh-462044, India; c Amity Institute of Environmental Sciences (AIES), Amity University Uttar Pradesh (AUUP), Noida-201313, India; d College of Biotechnology, Chaudhary Charan Singh Haryana Agricultural University (CCSHAU), Hisar 125004, India; e Department of Biotechnology, Graphic Era (Deemed to be University), Bell Road, Clement Town Dehradun, Uttarakhand-248002, India; f Department of Biotechnology, Deenbandhu Chhotu Ram University of Science and Technology, Murthal - Sonepat 131039 Haryana India; g Hansraj College, Department of Botany, University of Delhi, Delhi-110007, India; h Department of Botany, Banaras Hindu University, Varanasi-221005, India; i Division of Microbiology, ICAR-Indian Agricultural Research Institute, New Delhi-110012, India; j Department of Botany, K. S. Saket P. G. College, (Affiliated to Dr. Rammanohar Lohia Avadh University), Ayodhya, Uttar Pradesh, 224123, India; k Laboratoire CHEMBIOPRO (Chimie et Biotechnologie des Produits Naturels), ESIROI Agroalimentaire, Université de la Réunion, 15 Avenue René Cassin—CS 92003, Saint-Denis Cedex 09, 97744 La Réunion, France

**Keywords:** Bioremediation, Pesticides, Omics technology, Microbiome, Artificial intelligence

## Abstract

Chemical pesticides considered as one of the emerging environmental contaminants that severally affect the human health and soil and water ecosystem. Despite their well-documented adverse effects on fruit quality, soil structure, the emergence of pesticide-resistant pests, and human well-being, chemical pesticides are still widely used for crop protection, particularly in developing countries. Although to manage the chemical pesticides, various traditional approaches have been employed, however the higher cost, and the generation of toxic residues have shifted research attention toward eco-friendly and sustainable bioremediation strategies. Microorganisms including the bacteria, fungi, and algae play a crucial role in pesticide degradation by transforming toxic compounds into less toxic forms. However, to optimize microbial bioremediation, a comprehensive understanding of microbial metabolism and physiology is essential. In this context, omics technologies such as genomics, metagenomics, transcriptomics, proteomics, and metabolomics, offer powerful tools for elucidating the molecular mechanisms involved in pesticide degradation. These approaches facilitate the identification of microorganism, key genes, enzymes, and metabolic pathways responsible for the breakdown of pesticide compounds and their by-products. Furthermore, advanced technology like the gene editing can enhance the efficacy of pesticides biodegradation by knocking out undesirable genes or introducing beneficial ones in the microorganisms. The Artificial intelligence also plays a significant role in analysing big data, understanding microbial communities’ structure, identifying nature of pesticides and selecting or predicting the microbial species with enhanced pesticides degrading efficacy.

## Highlights

• Chemical pesticides pose serious risks to human health and environmental sustainability, especially in soil and aquatic systems.
• Microbial communities, including bacteria, fungi, and algae, offer promising, eco-friendly solutions for pesticide degradation and detoxification.
• OMICs technologies unravel microbial metabolic networks, enabling the discovery of key organisms and molecular components involved in pesticide breakdown.
• Emerging tools like gene editing and Artificial intelligence optimize microbial bioremediation by enhancing degradation efficiency and predictive accuracy.

## Graphical abstract

**Figure:**
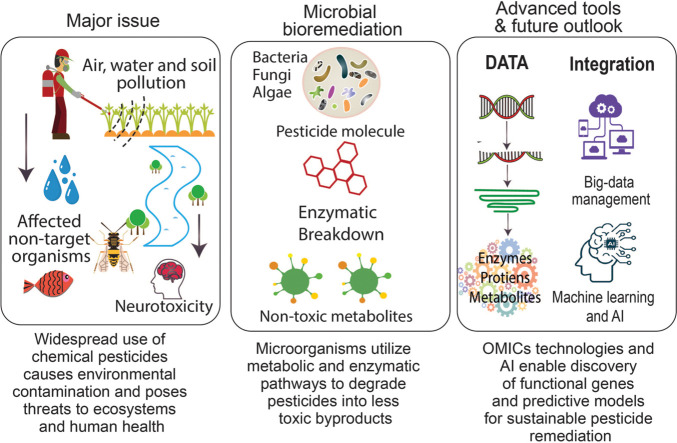

## 1 Introduction

**Fig. 1:**
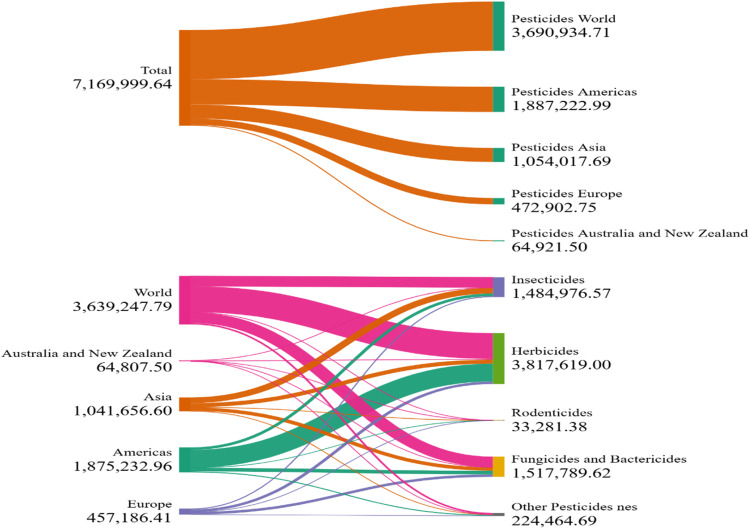
The global distribution and consumption of pesticides (value presented in tons) in selected countries (FAO STAT, 2024).

Pesticides are chemicals agents applied to various crops to protect them from insects, weeds, and pests, thereby enhancing agricultural productivity (Zhou et al., 2024). According to published report, the total consumption of pesticides in agriculture reached approximately 3.70 million tons in 2022, representing a 4 % increase compared to 2021 (FAO, 2024). Furthermore, the rate of pesticides consumption has increased by 13 % over the past decade and has nearly doubled since 1990. Fig. 1 illustrates the global distribution and consumption of pesticides across selected continent. Although chemical pesticides exhibit rapid and effective pest control responses, their long-term detrimental effects on soil health, fruit quality, native soil microflora, and human well-being are a major concern (Ahmad et al., 2024; Shekhar et al., 2024). Moreover, the emergence of pesticide-resistant pests has become a major challenge. According to Shattuck et al. (2023) and Lee and Choi (2020), only about 5 % of the total applied pesticides reaches to the target sites, while a large portion leaches out in to the soil or water, leading to environmental contamination. Furthermore, the bio-magnification and bioaccumulation of pesticides pose serious threat to the food chain integrity and are recognized as prime reason of health hazards (Tison et al., 2024).

**Fig. 2:**
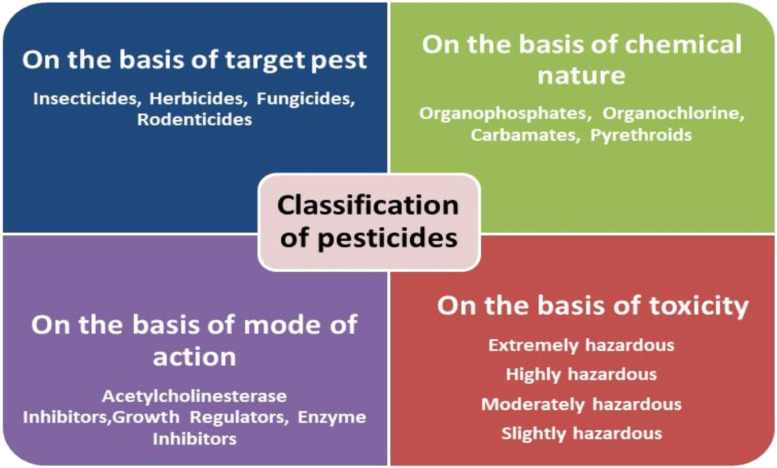
Classification of pesticides based on their targets, modes of action, chemical nature, and toxicity.

The negative consequences of pesticides generally depend on their concentration, chemical constituents and duration of exposure (Singh et al., 2022). Although pesticides are designed to act on a broad-spectrum level and primarily targets insects or pests within a specific area. They also adversely affect non-target organism such as animals, birds or humans by their adverse impact (Wan et al., 2025; Ahmad et al., 2024; Datta et al., 2025). Pesticides can be classified into four categories based on their target, mode of action, chemical natures, and toxicity as illustrated in Fig. 2. The chemical pesticides typically consist of active ingredients of chemical like organochlorine, organophosphate carbonates etc. that directly impact the target pest (Mitra et al., 2024). However, non-active or inert components such as surfactants solvents and aerosol, which did not show direct effect but help in emulsification, dilution or spraying on the target pest or sites (Lin et al., 2023). The long half-life, high bioaccumulation potential and significant toxicity, making the pesticides as persistent contaminants in food products such as vegetables, milk, and other consumables (Parra-Arroyo et al., 2022).

To remove or mitigate the challenges associated with pesticides contamination, several approaches such as, adsorption (Bose et al., 2023), steam extraction and thermal desorption (Dehghani et al., 2024), and mobilization (Kumar et al., 2022) are commonly employed. However, these physical, chemical and, thermochemical methods face significant limitations, including high operational costs, the release of toxic by-products, and the transfer of contaminants from one medium to another without actual degradation (Chakraborty et al., 2025; Chaudhary et al., 2024; Mitra et al., 2024; Leskovac and Petrović, 2023). In addition, environmental attributes such as temperature, pH, and moisture vary over time, allowing pesticides to desorb and percolate into groundwater. Moreover, the regeneration or disposal of used adsorbents produces secondary waste, complicating environmental management (Baskar et al., 2022).

Thermal desorption and steam extraction employ high temperatures or steam to evaporate pesticide residues from soil; however, these methods are energy-intensive and can disrupt soil structure, reduce organic matter, and destroy beneficial microbial communities (Lee et al., 2021). Such alteration may increase the mobility of residual pesticides, posing a higher risk of leaching. Moreover, incomplete volatilization and inefficient gas capture can also result in atmospheric contamination (Boonupara et al., 2023). AOPs, which generate hydroxyl (•OH) and sulfate (SO_4_^•−^) radicals to degrade pesticides, are extremely effective but have significant drawbacks. These include the potential formation of more toxic or mobile intermediate products, high operating costs and the leaching of intermediate compounds into soil and water systems (Gopalakrishnan et al., 2023).

Therefore, in recent years, various innovative technologies, particularly microbial based bioremediation has been employed for the effective and sustainable management of chemical pesticides (Aluffi et al., 2025; Fu et al., 2025). A wide range of microorganisms and their microbial products such as enzymes, biosurfactants, and biofilm aid in the environmental restoration by oxidizing, immobilizing, or degrading the pesticide residues (Pujiati et al., 2025; Fu et al., 2025).

Although the scalability and consistency of microbial based degradation of pollutant remain limited, despite the fact of promising laboratory outcomes. It has been generally observed that the efficacy of microbial pesticides degradation is often constrained by the fluctuating environmental conditions (Sharma et al., 2022). Under field conditions, variations in pH, temperature, and nutrient availability significantly influence microbial viability and their degradation potential. In addition, the introduction of non- native microbial species sometimes disturbs the native microbial community structure by causing competition with the native microbial species. The complex interaction of microbial species with the soil matrix, pollutants and the local biodiversity largely influence the efficacy and extent of microbial based pesticides bioremediation (Kebede et al., 2021; Pereira-Silva et al., 2025)

Although several reviews have described the adverse effects of pesticides on ecosystems and human health, as well as possible remediation strategies (Wu et al., 2025; Kumar et al., 2024; Chandrasekaran and Paramasivan, 2024), gaps still remain. For example, Rani et al. (2021) provided a general overview of pesticide effects and safe usage, discussed their impacts on human health, and outlined guidelines for their safe application in agriculture, the environment, and public health. However, El-Nahhal and El-Nahhal (2021) investigated the impact and consequences of pesticide residues in drinking water and potential removal strategies. However very few have critically evaluated the integration of microbial pesticides bioremediation with advanced technologies such as artificial intelligence (AI) and omics-based technologies. Addition to this combined application of AI, gene editing (CRISPR-Cas), RNA interference (RNAi), and omics technologies to optimize pesticide biodegradation remains underexplored.

Given the significant environmental implications of pesticides contamination impact of pesticides on environmental degradation, this review aims to explore their fate and their ecological and health impacts. It also highlights key knowledge gaps, microbial based pesticide biodegradation processes. Comprehensive frameworks on omics-based techniques, including transcriptomics, proteomics, metabolomics, and genomes, and their contribution in pesticide bioremediation. Finally, this review discoursed the emerging field of artificial intelligence (AI), and their significance in pesticides biodegradation and prospects for future research.

## 2 Fate of pesticides in the environment

Pesticides have the potential to disperse globally through atmospheric transport, making the atmosphere an important medium for their distribution. Pesticides may enter the atmosphere through drift application, vaporization post-application, or wind erosion of treated soil (Pathak et al., 2022). Pesticides and their breakdown products can travel long distances before being deposited on Earth's surface (Zhou et al., 2024). The fate of pesticides is influenced by several factors, including their entry, movement, and distribution across various environmental components such as soil, water, and air (Kumar et al., 2024). For example, when pesticides residues or contaminants enter to the sediments or aquatic ecosystem, they can impact entire trophic levels through biomagnification or bioaccumulation. Similarly, pesticide residues that leach into the soil or water can be reabsorbed by plant roots, re-entering the food chain and causing toxicity and adverse effects on humans and other organisms (Ahmad et al., 2024).

Furthermore, the fate of a pesticide after entering the soil is influenced by several factors such as soil biochemistry, the chemical or active agent of the pesticide, surrounding environmental conditions, soil organic matters and current meteorological conditions (Sarker et al., 2024). In addition, pesticides can infiltrate the hydrogeological environment, including groundwater, surface water, soil, and sediments, through various pathways such as agricultural runoff, industrial effluents, sewage discharge, and other commercial sources (Wu et al., 2023).

Moreover, physicochemical characteristics of pesticides such as chemical composition, volatility, water solubility, octanol-water partition coefficient, concentration, adsorption coefficient, half-life, and degradation potential influence the fate and movement of pesticides in aquatic environments (Zhou et al., 2024;).

## 3 Impact of pesticides

### 3.1 Impacts on soil and soil biological health

Soil is one of the most complex habitats, harbouring diverse microbial communities including bacteria, algae, fungi, protozoa. These microorganisms play an essential role in maintaining soil texture or productivity via different mechanisms like aeration, water permeability, nutrient recycling, and degradation of soil contaminants. According to studies one gram of soil contains approximately one million bacterial genomes (Prashar and Shah, 2016). Research has also shown that agricultural soils often contain a higher fungal biomass than bacterial biomass (Zelles et al., 1995) and that microbial necromass comprises of bacteria and fungus, account for about 1–4 % of total soil organic matter (Bahadori et al., 2021). While microbial metabolism plays a major part in the synthesis of soil organic matter, whereas microbial necromass contributes a minor amount (Cui et al., 2020; Bahadori et al., 2021). In addition, soil microbial species play pivotal role in plant growth promotion via following the mechanism like phytohoromone modulation, nutrient acquisition, phosphate solubilisation, etc. (Bolan et al., 2024; Bhanse et al., 2022).

**Fig. 3:**
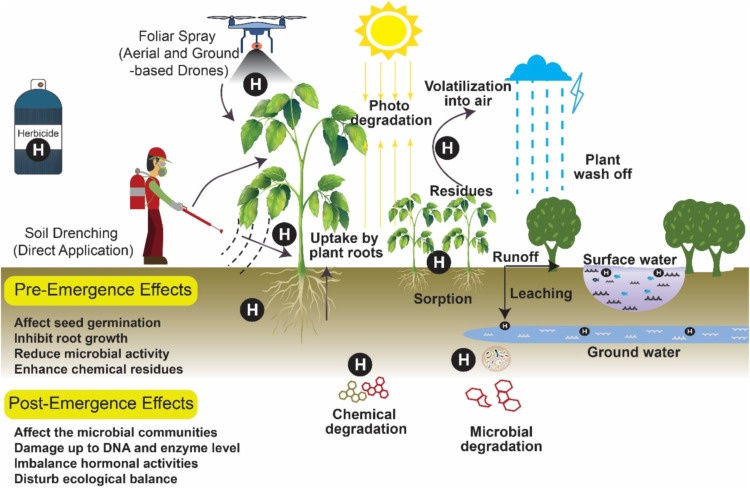
Overview of pesticides/herbicides exposure pathways and their mechanism, and, biological consequences.

To enhance agricultural productivity chemical pesticides or chemical fertilizers are frequently used; however, their extensive application adversely affects the environment by promoting algal blooms, fostering resistance in pathogenic microbes and pests, and distressing the soil biodiversity (Pathak et al., 2022). Previous studies have revealed that prolonged use of pesticides alters microbial community structure of soil leading to nutritional imbalances and reduced decomposition of soil organic matters (Sarker et al., 2024) and microbial diversity in the soil (Kumar et al., 2024) (Fig. 3). Xie et al. (2009) investigated the detrimental impacts of heavy metals copper (Cu) and cadmium (Cd) as well as the insecticide cypermethrin on soil microorganisms. Their findings indicated that microbial susceptibility to these pollutants was significantly higher in soils amended with synthetic fertilizers than in those amended with organic compost. Furthermore, pesticide applications negatively affect essential microbial functions such as nitrification, ammonification, and other biological activities, ultimately diminishing soil health and agricultural productivity (Zhou et a., 2024; Bhaskaralingam et al., 2025).

**Fig. 4:**
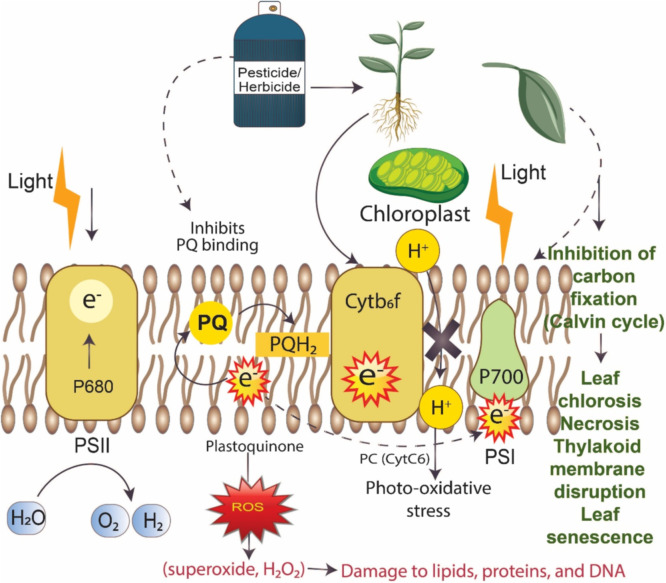
Schematic of herbicide/pesticides-induced disruption of the photosynthetic electron transport chain leads to ROS generation, photo-oxidative stress, and damage to cellular components.

The adverse consequences of the pesticides are largely attributed to the presence of specific chemical groups having high persistence and bioaccumulation potential (Rasool et al., 2022). The accumulation of pesticides in the environment adversely affects soil ecosystems by inhibiting natural nitrogen fixation and harming beneficial insects such as bees (Zhou et al., 2024; Singh et al., 2022). The pesticides application promotes the emergence of resistant pest, however reducing the microbial diversity of beneficial microorganism, which regulates the functioning of plant growth promotion or responsible to enhanced soil productivity (Cycoń et al., 2019). In a study Lu et al. (2018) observed that glyphosphate treatment, altered the diversity and relative abundance of beneficial microorganism and hamper their biological activity. Similarly, Fernandes et al. (2020) reported that the application of Atrazine adversely affected the functioning of ammonia-oxidizing bacteria and archaea. Pesticide use has also been linked to the emergence of antibiotic resistance genes (ARGs). For example, Xing et al. (2020) reported use of 2,4-D, carbaryl, and atrazine contribute antibiotic resistance genes (ARGs). Furthermore, the application of pesticides and herbicides disrupts the photosynthetic electron transport leading to the generation of reactive oxygen species (ROS), photo-oxidative stress, and cellular component damage, which collectively impair chloroplast function and leaf viability (Sule et al., 2022) (Fig. 4).

### 3.2 Impact of pesticides on human health

The pesticide comprises of various components, including chemical groups, specific active ingredients, and certain inert compounds that modulate its toxicity (Shekhar et al., 2024). The extent of pesticides toxicity depends upon several factors, such as formulation process, amount, and duration of exposure. However, in case of human beings the points of contact also influence the severity of pesticide contamination (Zhou et al., 2024). The inhalation or ingestion of elevated doses of pesticides can result in acute toxicity in the human beings; their prolonged exposure leads to in various chronic diseases and hormonal imbalances (Wu et al., 2025; Ahmad et al., 2024). For example, exposure of agriculturally utilized organophosphates and pyrethroids increased the probability of asthma in children living in proximity to agricultural fields (Mattila et al., 2021; Islam et al., 2023). Faria et al. (2014) previously reported incidence of lung cancer among individuals exposed to pesticides for more than two days per month. Extended pesticide exposure also negatively affects liver function by triggering overproduction of cytochrome P450 enzymes involved in detoxification, which in turn generates reactive metabolites that can cause liver damage (Liu et al., 2025). Published reports also revealed that pesticide exposure may impair the endocrine system, thereby increasing the risk of diabetes by influencing glucose metabolism and insulin resistance (Ahmad et al., 2024; Park et al., 2025). The dietary exposure to pesticides also results in the production of ROS and induces oxidative stress. Prolonged exposure to ROS can damage nucleic acids and exhibit mutagenic effects (Aguilar-Bañuelos et al., 2025).

**Fig. 5:**
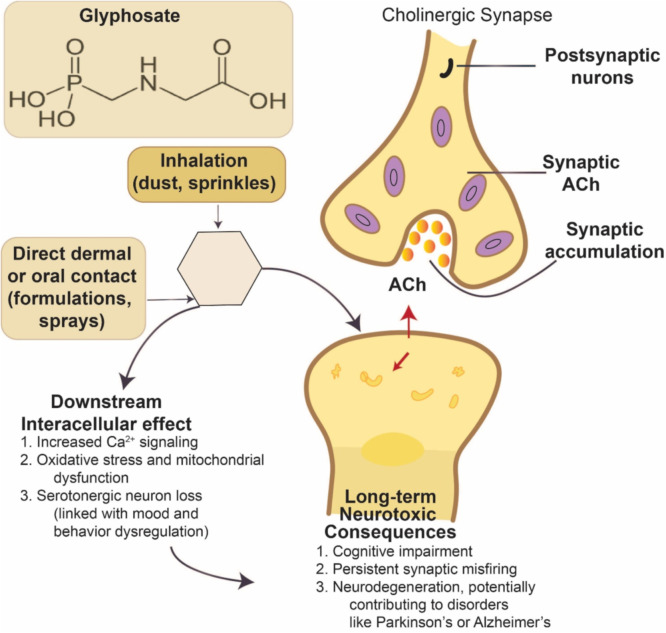
Schematic representation of glyphosate-based herbicide (GBH) neurotoxicity pathways. Based on findings from Avila-Vazquez et al. (2018), Ren et al. (2024), Madani & Carpenter (2022), and Duque-Díaz et al. (2022).

The continuous or the prolonged pesticides exposure may lead to various types of cancers, including breast, lung, and prostate cancer and neurodegenerative disorders (Asefa et al., 2024; Chilipweli et al., 2025). For example, Acetylcholinesterase is the enzyme that breaks down the neurotransmitter acetylcholine, and studies show that exposure to the pesticides carbamate and organophosphate can suppress this enzyme (Colovic et al., 2013). This inhibition can cause accumulation of acetylcholine and the overstimulation of cholinergic receptors, resulting in excessive salivation. Specific pesticides may interact with GABA receptors in the central nervous system, including organochlorines like indane and endosulfan. They can change the activation of GABA receptors, which can change inhibitory neurotransmission. Interruption of GABAergic signalling may lead to heightened lipid peroxidation and DNA damage in cerebral tissue, alongside neuronal hyperexcitability, seizures, and various neurological manifestations (Ahmad et al., 2024). Fig. 5 represent the glyphosate-based herbicide application and its neurotoxicity pathways.

## 4 Bioremediation of pesticides

For long-term environmental benefits, pesticide biodegradation is a novel approach to environmentally acceptable pesticide pollution control (Abuhasheesh et al., 2025; Magnoli et al., 2025; Liu et al., 2025a). Microorganisms are widely recognized for their beneficial roles in human welfare, particularly in the degradation of pesticides (Pathak et al., 2022; Fu et al., 2025; Aluffi et al., 2025; Kumar et al., 2024). During the process of bioremediation, microorganisms utilize pesticides as energy or carbon source (Bokade et al., 2023; Jia et al., 2023). Actinomycetes, algae, various bacterial and fungal strains, and others are examples of microorganisms which shows tremendous potential to degrade pesticides, which we have detailed below.

Algae are used in the bioremediation process known as "phycoremediation" to eliminate or degrade environmental contaminants, including pesticides (Abuhasheesh et al., 2025). By utilizing the capacity of algae to absorb, collect, or decompose toxic chemicals, it provides a sustainable and economical substitute for traditional technique (Nie et al., 2020; Kumar et al., 2020). Previously authors reported about microalgae role in pesticides bioremediation. For instance, Ni et al. (2014) reported that strains *Scenedesmus quadricauda* and *Microcystis* sp. significantly remove mesotrione and the efficacy was 18.3 % and 15.2 % respectively. Similarly, Wan et al. (2020) reported complete removal of trichlorfon by the use of strain *Chlamydomonas reinhardti.* Although the most mechanism followed by the microalgae strains during the management of pesticides contaminations are biosorption or bioaccumulation or biodegradation (Goh et al., 2022). During bioremediation, microalgal cells perform the process of oxido-reduction, hydrolysis or conjugation with the cellular metabolites.

Additionally, the microalgae are photosynthetic organisms releases the oxygen which directly or indirectly play essential role in metabolic process of microalgae and coverts the pesticides into the organic matter or biomass (Hussein et al., 2017; Akmukhanova et al., 2025). Mojiri et al. (2022) investigated the ability of *Chlorella vulgaris* to remove cypermethrin and chlorpyrifos in a concentration of 0.32 mg L⁻¹, reporting impressive reductions of 88.80 % for chlorpyrifos and 93.12 % for cypermethrin after 69.7 h of treatment. This effectiveness is further supported by Nanda et al. (2019), who found that *C. sorokiniana* achieved a 90 % removal of malathion (25 μg L⁻¹) within 14 days in the cultured medium of Bold’s Basal Medium (BBM). Kurade et al. (2016) reported 94 % removal efficacy of *C. vulgaris* against the pesticides diazinon in the time frame of 12 days. Similarly, Castellanos-Estupiñan et al. (2022) reported about *Chlorella* sp., *Scenedesmus* sp., and *Hapalosyphonsp* species having the 50, 75 and 10 % removal efficacy of chlorpyrifos respectively. Previously authors reported about the various microalgae strains, which had been successfully used to remove or degrade the pesticides (Table 1).

**Table 1:** Summary of the role of microbial species in pesticides bioremediation.

Microbial species	Pesticides	Observation	References
*Microalgae*			
*Chlorella sorokiniana*	Malathion (100 ppm)	Tolerance of 100 ppm	Nanda et al., 2019
*C. vulgaris*	Diazinon (25 mg L^−1^)	95.2 % in 12 days	Ianelly and Elsa, 2024
*C. vulgaris*	Atrazine (0.002 mg L^−1^)	89.2 % in 0.0417 days	Hussein et al., 2017
*S. platensis*	Malathion (0.02–100 mg L^−1^)	54 % in 20 days	Ibrahim et al., 2014
*C. mexicana*	Diazinon (20 mg L^−1^)	64 % in 12 days	Kurade et al., 2016
*Scenedesmus intermedius*	Lindane	75 %–98 % degradation	González et al. 2012
*Chlamydomonas* *reinhardtii*	Prometryne	100 % in 6 days	Jin et al. 2012
*Bacterial strains*			
*Pseudomonas* genus B3	Profenofos and chlorpyrifos groups	Chlorpyrifos 63 % and profenofos 67 % degradation	Pujiati et al., 2025b
*Pseudomonas sp*.	Ethiprole	79.7 % within 24 h for a 50 mg L^-1^ ethiprole solution.	Wei et al., 2025
*Bacillus cereus, Paenibacillus lautus*	Malathion, chlorpyrifos, coumaphos (80 mg L^−1^)	52.4 %, 78.8 % and 79.5 %, within 12 days	Rodríguez-Orozco et al., 2025
*Bacillus* strains (I3 and I6)	Hexachlorobenzene	11.5 ± 1.47 % and 21.1 ± 0.84 % respectively	Mota et al., 2025
*Bacillus* consortium	Chlorpyrifos (100 ppm)	91 % within 6 days	Varghese et al., 2024
*Bacillus spizizenii*	Lambda cyhalothrin	80.79 %	Ranade et al., 2024
Bacillus subtilis	dimethoate (0–80 µg L-^1^)	100 % within 10 days	Shalaby et al., 2024
*Bacillus* sp.	λ-cyhalothrin	100 % within 96 h.	Umar et al., 2024
*Stenotrophomonas maltophilia*	Monocrotophos	Tolerance of 1000 ppm	Harshitha et al., 2024
*Stenotrophomonas maltophilia* RNC7	Chlorpyrifos (100 mg L^−1^)	77.1 % degradation within 5 days.	Bhende et al., 2024
*Bacillus cereus*	Imidacloprid (0.03 mM)	92 % within 11 days	Talpur et al., 2023
*Rhodococcus Bacillus*	DDT	85 %	Mudziwapasi et al., 2016
*Bacillus* sp. KF984414	Endosulfan	74 % in broth and 67 % in soil	Rani and Kumar, 2017
*Bacillus* sp. LN849696	Endosulfan	70 % in broth and 63 % in soil	Rani and Kumar, 2017
*Pseudomonas* sp.	Endosulfan	70–80 %	Zaffar et al., 2018
*Kocuria assamensis*	Chlorpyrifos and malathion	71.3 % and 85 % respectively	Mehta et al., 2021
*Pseudomonas rhizophila*	Pentachlorophenol		Hassen et al., 2018
*Engineered Pseudomonas putida* KT2440	Organophosphates and pyrethroids	100 %	Zuo et al., 2015
*Bacillus aryabhattai*	Parathion	56 %	Pailan et al., 2015
*Bacillus thuringiensis*	Chlorpyrifos	(30.34 %) was observed in soil supplemented with BtS.	Aceves-Diez et al., 2015
*Bacillus* sp. DG-02	Fenpropathrin	93.3 % of 50 mg L^–1^ fenpropathrin	Chen et al., 2014
*Bacillus firmus*	Fipronil	100 % after 35–42 days	Mandal et al., 2014
*Fungal strains*			
*Aspergillus Niger*	Malathion	29 % and 68 %, in wild and malathion tolerant *Aspergillus Niger* after 5 days	Mohapatra et al., 2024
*Aspergillus fumigatus*	Ametoctradin	71 % within 16 days	Majid et al., 2024
*Aspergillus flavus*	Profenofos and Chlorpyrifos	36 % chlorpyrifos and 81 % profenofos	Pujiati et al., 2025
*Penicillium citrinum, Aspergillus fumigatus, A.terreus, Trichoderma harzianum*	Chlorfenvinphos		Oliveira et al., 2015
*B. laricina* JAS6+ *A. tamarii* JAS9	Endosulphan (1000 mg L^−1^)	100 % within 4 days	Abraham and Silambarasan 2014
*Penicillium* sp.	Endosulfan	100 % in 6 days	Romero-Aguilar et al., 2014
*Phanerochaete chrysosporium*	Chlorpyrifos (50 mg L ^-1^)	100 % in 6 days	Barathidasan et al. 2014
*Trametes versicolor*	Imiprothrin (20 mg L^−1^)	>90 % within 2 days	Mir-Tutusaus et al. 2014
*Gloeophyllum trabeum*,	DDT	89 % in 14 days	Purnomo et al. 2008
*Aspergillus terreus*	Chlorpyrifos (300 mg kg^−1^ soil)	100 % within 24	Silambarasan and Abraham, 2013
White rot fungus	Lindane (y-HCH).		Quintero et al., 2008
*Boletus edulis, Gomphidius viscidus, Laccaria bicolor*, and *Leccinum scabrum*.	DDT (5 mg L^−1^)	100 %	Huang et al., 2007
*Phanerochaete chrysosporium*	Atrazine and Alachlor		Chirnside et al., 2011
*Trametes hirsuta*	10 μM Endosulfan	>90 % in 42 days	Kamei et al., 2011
*Coriolus versicolor, Hypholoma fasciculare Stereum hirsutum*	Diuron, Metalaxyl, Atrazine or Terbuthylazine	86 %, diuron, atrazine and terbuthylazine in 42 days while metalaxyl <44 % in the same time interval	Bending et al., 2002
*Aspergillus niger*	Beta‐endosulfan		Mukherjee and Gopal., 1994

Among different biological methods, the pesticide degradation using bacteria are much preferred because of ease in cultivation and rapid growth (Nie et al., 2020). The important bacterial species employed for the elimination and degradation of pesticides belong to the genera *Rhizobium, Pseudomonas, Bacillus, Acinetobacter, Agrobacterium, Micrococcus, Xanthomonas, Klebsiella, Burkholderia, Lactobacillus, Staphylococcus, Acromobacter, Enterobacter*, and *Pantoea* (Tanveer et al., 2024; Liu et al., 2025b) (Table 1). Chandrasekaran and Paramasivan (2024) have recently reviewed the role of PGPB in not only the degradation of pesticides of varied nature, but also in the improvement of plant growth attributes. The PGPB were shown to degrade noxious pesticides such as endosulfan, phorate, malathion, and carbofuran. Some of the noticeable bacterial genera with remarkable contribution towards pesticide degradation and removal include *Klebsiella* sp. *Ochrobactrum intermedium, Acinetobacter* sp. PDB4, *Bacillus* sp., etc. (Li et al., 2020; Rani et al., 2019; Kosimov et al., 2024; Mota et al., 2025). In a study, Jia et al. (2021) reported the of atrazine removal efficacy of strain *Paenarthrobacter* sp. AT-5, which showed 95.9 % removal efficacy of 5 mg kg^−1^ atrazine within 7 days. Cheng et al. (2024) reported that *Bacillus cereus* strain DC-1 exhibited a 99.23 % degradation efficiency for butachlor (100 mg kg⁻¹) in soil within 12 days. The removal as well as degradation of pesticide chlorpyrifos by the biomass of bacteria identified as *Pseudomonas stutzeri* using 16S rRNA sequencing, amalgamated with the agro-waste derived from *Delonix regia* seeds, has been emphasized by Saravanan et al. (2020). The combined application of the biosorbent was demonstrated to eliminate 95.29 % and 93.10 % pesticide from water and soil, respectively by the end of two weeks treatment, advocating implications in the management of pesticide affected sites. Yang et al. (2024) have suggested the potential of biochar immobilized *Acinetobacter* YH0317 in the successful elimination of 80.42 % pesticide bensulfuron-methyl from paddy field, in comparison to individual application of boron doped biochar and bacterium in question. The greater removal efficiency of pesticide by biochar immobilized bacterial strain was ascribed to improved activity even in the unfavourable and complex environmental conditions rendered by protection of selected bacterium. The experimental investigation on degradation and removal of pesticides including lactofen, acetamiprid, and carbendazim by bacterial species *Bacillus* sp. *Pigmentiphaga* sp. and *Rhodococcus* sp. respectively has been demonstrated by Zhao et al. (2025). The degradation potential of target pesticides by the selected immobilized bacterial strains was registered to be >70 % under defined conditions of 30 °C temperature, pH7, and 6 % inoculum after 48 h of duration. Approximately absolute removal of the pesticides was achieved in the aqueous medium during one week treatment. The immobilized bacterial strains were able to degrade abovementioned pesticides introduced in water and soil, thereby suggesting plausible role in addressing the challenges of agricultural and environmental safety compromised by diverse contaminants.

Endophytic microorganisms living within diverse host plants are well acknowledged to hold the potential of pesticide degradation and detoxification in terrestrial environment (Murthy, 2025). The bacterial species of genera *Pseudomonas* and *Bacillus* briefly reported for pesticides bioremediation including atrazine and alachlor into less toxic derivatives as compared to other microorganisms (Mesquini et al., 2015). The bioremediation of pesticide-contaminated sites by bacteria is influenced by multiple factors. These include the concentration and chemical nature of the pesticide, pH, temperature, and the number of selected bacterial strains. The physiological state of the bacteria whether free or immobilized along with their tolerance, nutritional conditions, and exposure duration also plays a key role. In addition, interactions with other soil microorganisms and the associated enzymatic activities affect the degradation efficiency. Therefore, optimizing these parameters is essential before translating the process to field conditions. (Chia et al., 2024; Liu et al., 2024; Arias-Castro et al., 2025). A single bacterial strain may not show efficiency in the degradation and remediation of all kinds of pesticides; therefore, selection of the best suited bacterium or consortia thereof could be considered for management of pesticide affected sites. Additionally, understanding detailed biochemical and molecular mechanisms apart from the different statistical approaches such as design of experiment (DOE) and response surface methodology (RSM) based methods for improving the degradation of target pesticides can be achieved (Malla et al., 2023; Chen et al., 2025; Wu et al., 2025).

Degradation of pesticide by fungi is called as “mycoremediation” and regarded as valuable tool for pesticide degradation through large array of interaction causing structural modification and degradation of pesticide molecules (Pinto et al., 2012; Prajapati et al., 2022; Bhandari, 2017; Aluffi et al., 2025; Moubasher et al., 2025; Pujiati et al., 2025) (Table 1). Fungi have superiority over other microorganisms because of large mycelia networks, compromised specificity of enzymes, and the use of target pesticide as growth substance, favouring biodegradation process (Maqbool et al., 2016; Dinakarkumar et al., 2024) and contributing towards the nutrient cycling. Further, the development of tolerance upon exposure to pesticide to be degraded, growth on diverse waste substrates, high survivability even at different temperature and pH rendered by spore formation, easy cultivation under laboratory condition, better reproductive abilities, large surface area, integration with other available methods showed their suitability for pesticides mycoremediation (Bhattacharya et al., 2020). In a study, Oliveira et al. (2015) evaluated the pesticides degradation potential of selected fungal species. The introduction of fungal species such as *Aspergillus terreus A. fumigatus, Penicillium citrinum* in aquatic media depicted degradation of chlorfenvinphos to undetectable level, however, no degradation was registered for isoproturon, atrazine, and diuron. The degradation of pesticide chlorpyrifos by two different fungal strains identified as *Byssochlamys spectabilis* and *Aspergillus fumigatus* tolerant to 600 mg L^‒1^ concentration, and recovered from waste soil has been demonstrated by Kumar et al. (2021).

The experimental investigation conducted by Hu et al. (2022) reported degradation potential of *Trametes versicolor* against wide range of pesticides including malathion, acetamiprid, and imidacloprid. The treatment with *Trametes versicolor* for 48 h facilitated complete elimination of malathion (1 mg L^‒1^) however, acetamiprid and imidacloprid with the concentration 4 mg L^‒1^ was recorded to be 20 % and 64.7 %, respectively within 7 days of treatment. The analysis of enzymatic degradation of pesticide suggested seven, two, and one transformation product in solution phase derived from malathion, imidacloprid and acetamiprid, respectively. The implications of catalytic activities of oxidoreductases, particularly from filamentous fungi like *Trametes, Phanerochaete, Trichoderma*, and *Pleurotus* in directing the efficient degradation of pesticides (Roy et al., 2025). The filamentous fungi are acknowledged to synthesize a wide range of oxidoreductases enzymes such as cyrochrome P450 monooxygeneses, tyrosinases, laccases, and peroxidases which offer significant potential for remediation of pesticides that poses risk to human health and environment (Lin et al., 2022; Wang et al., 2025a; Wang et al., 2025b; Yin et al., 2025).

The experimental investigations carried out by Cavalcante et al. (2025) has revealed the efficiency of different mixed culture comprising of different strains of *Aspergillus niger, Trametes versicolor*, and *Phanerochaete chrysosporium* in the removal of herbicide paraquat. The laboratory result demonstrated better performance of mixed culture designated as CI, pointing towards the suitability of test fungal species in bioreactor systems without the dependency on co-substrate for the removal of pesticides. Thus, the diverse fungal species could have immense opportunities in managing the hazardous impacts of synthetic pesticides. The real translation of capabilities of isolated fungi for the degradation and remediation of pesticides would require extensive investigation under varied environmental conditions.

Further, the isolation, purification, and immobilization of pesticides degrading fungal enzymes are considered as an additional step towards environmental clean-up processes (Xie et al., 2010; Bebić et al., 2020; Serbent et al., 2024; Ulu et al., 2024). Recently, the pivotal role of different entomopathogenic and filamentous fungi including *Metarhizium robertsii, M. anisopliae, Beauveria species, Pleurotus ostreatus*, and *Phanerocharte chrysosporium* in the degradation of pesticides present in agro-ecosystem has been reviewed by Swathy et al. (2024), indicating sustainable management of pesticide contaminated sites. Previous published study suggested integration of different strategies relying on traditional and omics approaches such as transcriptomics and metagenomics, apart from system biology, in the management of pesticides posing undesirable consequences on human and environmental complexes (Ali et al., 2025).

The efficiency of microbial strains in pesticide degradation is largely context-dependent rather than universally effective, as it varies with the nature and concentration of the pesticide, environmental factors, and microbial adaptability. Previous published report demonstrated some bacterial genera, such as *Pseudomonas, Bacillus*, and *Streptomyces*, exhibit broad-spectrum degradation potential, while their performance fluctuates across soil types, pesticide formulations, and physicochemical conditions (Shah et al., 2024; Ahmad et al., 2022). These inconsistencies are reported in the previous studies due to the variations in experimental design, environmental factors, pesticide concentration, inoculum density and assessment duration (Elshikh et al., 2022). For instance, *Pseudomonas putida* strain (epl) has been reported to effectively degrade ethoprophos- a organophosphate at the temperature of 20 and 35 °C and at soil water potentials of −33 and −10 kPa. However, degradation potential significantly reduced at 5 °C or at a soil water potential of −1500 kPa. In addition, the degradation potential of ethoprophos- was optimum in the soil at the pH range of 6.8 and 8.3, while at the soil pH 5.4, complete loss of degradation potential was recorded (Karpouzas and Walker, 2000). In a study Elshikh et al. (2022) evaluated the pesticides degradation potential of bacterial strains *B. cereus* CP6 and *K. pneumoniae* CP19, which degraded >70 % chlorpyrifos at 200–300 mg/L initial concentrations, and the variation in concentration of pesticides influences the pesticides degradation potential.

In a study, Zhang et al. (2024) reported the biochemical pathways and degradation efficiency of glyphosate using a microbial consortium, YS622, comprising *Azospirillum, Cloacibacterium*, and *Ochrobacterium* genera. The consortium achieved 100 % degradation of 50 mg L⁻¹ glyphosate within 36 h. Additionally, consortium YS622 demonstrated 97 % degradation of 60 mg L⁻¹ glyphosate within 36 h in both sterilized and unsterilized water–sediment systems. In another study, Bourguignon et al. (2014) found that *Streptomyces* sp. A14 exhibited excellent growth and methoxychlor (MTX) removal in liquid culture, achieving 100 % degradation within 24 h at 1.66 mg L⁻¹ MTX. However, in soil microcosms, *Streptomyces* sp. A14 removed 40 % and 76 % of 8.33 and 16.60 mg kg⁻¹ MTX, respectively, after 28 days of incubation. The strain degraded MTX through dechlorination, dehydrogenation, and CN-replacement, producing multiple intermediates metabolites. Góngora-Echeverría et al. (2020) assessed the degradation efficiencies of multiple pesticides (2,4-D, atrazine, carbofuran, diazinon, and glyphosate) using both a microbial consortium and individual bacterial strains in liquid media. The consortium, comprising *Ochrobactrum* sp. DGG-1–3, *Ochrobactrum* sp. Ge-14, *Ochrobactrum* sp. B18, and *Pseudomonas citronellolis* ADA-23B, successfully degraded >90 % of atrazine, carbofuran, and glyphosate, whereas 2,4-D and diazinon exhibited higher persistence. Nonetheless, the consortium demonstrated superior degradation performance for 2,4-D, carbofuran, and diazinon (DT₅₀ < 10 days) when compared with the individual pure strains.

Jariyal et al. (2015) isolated and characterized *Brevibacterium frigoritolerans* strain Imbl 2.1 as a novel phorate-degrading organism. In soil bioaugmentation experiments, this strain exhibited exceptional degradation capacity, achieving 89.81–92.32 % removal of phorate in agricultural soils amended with 100–300 mg/kg of the pesticide. In contrast, non-inoculated control soils showed only 33.76–40.92 % natural attenuation over the same experimental period. In a study Zhang et al. (2022) reported atrazine degradation efficacy of strain *Paenarthrobacter ureafaciens* ZF1 through miniature experiments. The strain ZF1 showed 100 % degradation efficacy of 100  mg L^− 1^ atrazine within 2 h in liquid medium while showed 99.3 % degradation of 100  mg kg^− 1^ atrazine in soil within 6 days. In a study, Fuentes et al. (2017) reported the simultaneous removal of a mixture of three organochlorine pesticides lindane, chlordane, and methoxychlor from different soil systems using a native *Streptomyces* consortium. In liquid culture, the consortium exhibited optimal growth and achieved removal efficiencies of 40.4 % for lindane, 99.5 % for methoxychlor, and 99.8 % for chlordane. In sterile soil microcosms, the consortium was able to grow across different soil textures (clay silty loam, sandy, and loam) without significant variation. However, in non-sterile clay silty loam soil, the consortium removed only 11 % of lindane, 20 % of methoxychlor, and 5 % of chlordane. In sterile clay silty loam soil slurries, removal efficiencies were 12.5 % for lindane, 10 % for chlordane, and 26 % for methoxychlor. These results indicate that the pesticide removal efficiency of the *Streptomyces* consortium is strongly influenced by soil texture and sterility conditions.

These discrepancies emphasize the necessity of a robust and standardized experimental design under realistic or varying environmental conditions for effective pesticides degradation. The practical recommendation for sustainable microbial based pesticides remediation includes various methods (Kuppan et al., 2024). For example, improvement in soil organic matter through application of compost, biochar, crop residues, that enriches microbial diversity and the metabolic activity of the microbes (Ali et al., 2025; Das et al., 2025). Mulching and crop covering for the buffering of soil temperature to provide a stable microenvironment for the microbial activity (Kong and Six 2012; Tian et al., 2022). And the maintenance of the neutral pH for the bacterial species and slightly acidic pH for the fungal species by adding lime to the acidic soil and gypsum to the alkaline soil, ensure optimum microbial enzyme activity for the efficient pesticides degradation. In addition, improving soil structure and aeration through reduced tillage and the use of organic amendments enhances oxygen flow, supporting aerobic degradation processes (Qian et al., 2025).

## 5 Mechanism and factors governing the bioremediation of pesticides

### 5.1 Mechanisms

The microorganisms are acknowledged to employ a large array of biological processes to eliminate and degrade the hazardous pesticides. Biosorption, transformation, and mineralization are considered as an important mechanism responsible for pesticide bioremediation (Kumar and Sachan, 2021). Experimental investigations have demonstrated the synthesis of enzymatic systems including different dioxygenases such as extradiol dioxygenase, and aromatic ring-breaking dioxygenases as well the surfactant by the bacterium *P. rhizophila* S211 having characteristic contribution in the removal of selected pesticide (Hassen et al., 2018). The dioxygenases are recognized to have a pivotal role in modulating recalcitrant pesticides into intermediate compounds or metabolites entering to major degradation pathways. The breakage of ester bond of carbendazim with the resultant formation of methyl formate and 2-aminobenzimidazole followed by transformation to different compounds has been proposed as mechanism of degradation by Bhandari et al. (2022). The pesticide degradation involves different processes like hydrolysis, synthesis, condensation, dehydrogenation, oxidation, and reduction and it varies with the types or chemical constituents of pesticides and nature of microorganisms (Kumar and Sachan, 2021). The detailed mechanism of biodegradation of pesticide by lactic acid bacteria has been illustrated recently by Kiruthika et al. (2025). Adsorption and biodegradation have been identified as the primary mechanisms involved in the breakdown of organophosphate (OP) pesticides. Many bacterial systems utilize these pesticides as sources of carbon and nitrogen, a process facilitated by diverse enzymatic activities including carboxylesterases and phosphatases which play key roles in catalyzing OP hydrolysis and subsequent mineralization.

During microbially mediated bioremediation, various metabolites are generated as intermediate degradation products. Some of these intermediates may not be further degraded because of their toxic nature, which can inhibit the degrading microorganisms (Fang et al., 2019). Therefore, effective pesticide biodegradation requires careful selection of competent microbial strains, identification of appropriate metabolic pathways, and a thorough understanding of the toxicity and persistence of intermediate metabolites (Huang et al., 2021; Farhan et al., 2021).

Previously various authors reported the detailed degradation pathways, intermediate compounds and the role of microorganisms in effective pesticides degradation. Mali et al. (2022) elucidated the degradation pathway of chlorpyrifos (CP) and identified 3,5,6-trichloro-2-pyridinol (TCP), 2,6-dihydroxypyridine (DHP), and diethyl‑thio-phosphoric acid (DETP) as key intermediate metabolites. Treatment with *Arthrobacter* sp. HM01 demonstrated the strain’s ability to degrade CP through the action of an organophosphate hydrolase (opdH) enzyme. This enzyme cleaves the ester bond in CP, leading to the formation of TCP and DETP, which are subsequently metabolized into non-toxic end products such as DHP, phosphoric acid, and pyruvic acid (Mali et al., 2022). Similarly, during the degradation of aldrin, dieldrin is formed as a major intermediate metabolite. Aldrin degradation proceeds through three primary pathways: oxidation, reduction, and hydroxylation. In contrast, the degradation of dieldrin involves four pathways oxidation, reduction, hydroxylation, and hydrolysis resulting in major metabolites such as 9-hydroxydieldrin and dihydroxydieldrin. In the oxidative degradation pathway, the non-chlorinated double bond of aldrin undergoes epoxidation as the initial metabolic step, converting aldrin into dieldrin. When this non-chlorinated double bond is further oxidized, microorganisms metabolize aldrin into dihydrochlordenedicarboxylic acid and monohydroxy-dihydrochlordenedicarboxylic acid (Bandala et al., 2006; Pang et al., 2022) The microorganisms, such as *Pseudomonas fluorescens, Trichoderma viride, Pleurotus ostreatus, Mucor racemosus*, etc. have the capability to degrade the dieldrin (Pang et al., 2022).

In a study, Feng et al. (2020) reported the degradation pathways and specific intermediate metabolites formed during the breakdown of strobilurin fungicides. The general degradation process for strobilurins, such as benzene kresoxim-methyl, involves initial oxidative cleavage or hydroxylation of the acrylate double bond, leading to the formation of BKM-enol or azoxystrobin-enol. Subsequent hydrolysis or dealkylation steps occur, including cleavage of the methyl ester group or removal of alkyl substituents. These intermediate compounds then undergo further metabolic transformations, such as secondary oxidative cleavage and decarboxylation. During microbially mediated bioremediation of strobilurins, microbial strains including *Bacillus, Pseudomonas*, and *Klebsiella* species facilitate carboxylester hydrolysis through the secretion of esterase enzymes, thereby accelerating the breakdown of these fungicides. Similarly, in another study, Zhang et al. (2022) reported the glyphosate-degrading potential of *Chryseobacterium* sp. Y16C. The strain efficiently degraded 400 mg·L⁻¹ of glyphosate within 4 days and exhibited a maximum specific degradation rate of 0.91459 d⁻¹. It was capable of degrading glyphosate via the aminomethylphosphonic acid (AMPA) pathway and also demonstrated the ability to tolerate and degrade AMPA at concentrations up to 800 mg L⁻¹. Generally, microbial degradation of glyphosate proceeds through either the AMPA pathway or the sarcosine pathway. In the AMPA pathway, the C–N bond of glyphosate is cleaved by an oxidase enzyme, yielding AMPA and glyoxylate. The glyoxylate is subsequently metabolized through the tricarboxylic acid (TCA) cycle, while AMPA is further degraded to support microbial growth. In contrast, the sarcosine pathway involves C–P lyase–mediated cleavage of the C–P bond, producing sarcosine and inorganic phosphorus ( Castrejón-Godínez et al., 2021). In this context, identifying the specific metabolic pathways, intermediate products, and appropriately selecting efficient microbial strains is crucial for effective pesticide degradation (Duc and Oanh, 2025; Hu et al., 2025).

### 5.2 Governing factors

The degradation of pesticides by the microbes depends upon various factors including temperature, pH, moisture content, soil characteristics, availability of oxygen, composition of media, microbial species, nature and concentration of pesticide etc. (Levio-Raiman et al., 2021; Carranza et al., 2025; Roy et al., 2025). These factors play crucial role in the pesticide degradation mediated by microbial species including bacteria, fungi, microalgae etc. As the survival, growth and metabolic activity of the microorganism dependent upon the temperature, and at particular pH, therefore, it directly influences the microbial efficacy of pesticides degradation (Doolotkeldieva et al., 2018; Roy et al., 2025).

Temperature influences the metabolic activity and enzymatic activity of the microorganisms, which is essentially required for the pesticide’s degradation. The higher temperature can denature the enzymes and even survivality of microorganisms, while the lower temperature slow down microbial activity and restrict the microbial efficacy of pesticide degradation (Reedich et al., 2017; Chia et al., 2024a). Similarly, the soil pH affects the microbial community structure and functional attributes of microbial enzymes. Studies showed that the most bacterial strains preferred neutral pH for optimum pesticides degradation, while slightly acidic pH favoured by fungal species (Singh et al., 2003). The variation in soil pH not only affect the viability of microorganisms but also affect enzymatic activity of microorganisms, which are required for effective pesticides bioremediation (Andleeb et al., 2016; Doolotkeldieva et al., 2025). Additionally, the pH alters the chemical stability and solubility of pesticides, which influence the pesticides accessibility to microbes. Previous published report showed the impact of different factors on the pesticide’s degradation. For example, rate of chlorpyrifos degradation increased at near-neutral pH and moderate temperature, while acidic and higher alkaline soils slow microbial turnover (Singh and Walker, 2006; Gonzales-Condori et al., 2020). Magnoli et al. (2025) investigated the impact of pH and temperature on the growth and removal of pesticide by two different strains of *Penicillium crustosum*. The optimum growth and higher efficacy of pesticides degradation by both fungal strains were recorded at pH 7 and temperature 25 °C (Magnoli et al., 2025). Recent isolation and optimization studies report that bacterial and fungal pesticide degraders typically exhibit optimal temperature and pH ranges—for example, many chlorpyrifos- and other organophosphate-degrading strains perform best at 20–37 °C and pH 6–8. Although some strains tolerate conditions outside these ranges, their degradation activity generally declines under suboptimal conditions (Mali et al., 2022; Wepukhulu et al., 2024).

Soil properties such as texture, moisture, organic matter content and nutrient availability largely affect native microbial community’s structure (Philippot et al., 2024). The variation in the soil properties, or the accumulation of higher concentration of the pesticides can negatively affect the active microbial populations and affect their pesticides degradation efficacy. Adequate moisture is essential for microbial metabolism. The moisture level affects the binding affinity and mobility of pesticides, thereby affecting their bioavailability and degradation rate by microorganisms (Philippoussis et al., 2001; Bhatt et al., 2021; Sun et al., 2024). The availability of water influences oxygen transportation, that in turn modulates the growth and synthesis of enzymes by a particular microbial species. The extreme dryness or waterlogging can suppress microbial activity by altering oxygen availability (Philippoussis et al., 2001).

Supplementation of carbon and nitrogen sources in the medium has been illustrated to modulate the degradation of pesticides by fungi. Amalgamation of glucose to 1 % in the medium causing optimum degradation of methamidophos by the fungus *Penicillium oxalicum* ZHJ6 is reported by Zhao et al. (2010b). Improved degradation of dieldrin under varying concentrations of carbon source supplied in the form of glucose, and nitrogen by *Mucor racemosus* has been deciphered by Kataoka et al. (2010). Additionally, the complexity, functional groups or the nature of pesticides like solubility, volatility and the persistence largely determined the biodegradation efficacy.

Further, pesticides having the recalcitrant structures, such as organochlorines (e.g., DDT), often resist microbial degradation because of the strong and stable bonds within their molecular framework (Singh and Walker, 2006). Additionally, the concentration especially the higher load of pesticides also plays detrimental role on microbial cell or microbial activity, thus negatively impact pesticides biodegradation (Cycoń et al., 2017). The hydrophobicity, solubility of pollutants also negatively impacts the microbial based degradation of pesticides (Bhatt et al., 2021).

The selection, characterization, survivability, and metabolic activity of microbial strains are key factors that determine the rate of pesticide degradation. However, in complex soil environments, synergistic interactions among different microbial strains often enhance degradation through metabolic cooperation or cross-feeding (Kumar et al., 2024). As the optimization of diverse parameters affecting the degradation of pesticide, therefore, needs to be considered essentially for addressing the rising concerns of pesticides posing risk to human health and the environment.

## 6 Exploring microbial degradation through omics-based approaches

Microbial interventions have emerged as a promising tool for the soil remediation. Despite the success of these biological approaches, significant gaps persist in comprehending the complicated mechanisms and environmental attributes that impact bioremediation efficiency (Olu-Arotiowa et al., 2019). One of the primary limitations is that a substantial proportion of microorganisms present in natural ecosystems remain unculturable under conventional laboratory conditions, thereby restricting their potential uses in biotechnological and environmental field. However, with the rapid advancement of high-throughput sequencing technologies, researchers can now delve into the functional genomics of environmental microbial communities. These technologies facilitate the identification and characterization of degradation-related genes and pathways that are responsible for the breakdown of recalcitrant pollutants, even in microbes that are not amenable to culturing (Bhatt et al., 2021). This advancement has greatly enhanced our insight into the metabolic potential of microbes and their roles in contaminated ecosystems.

**Fig. 6:**
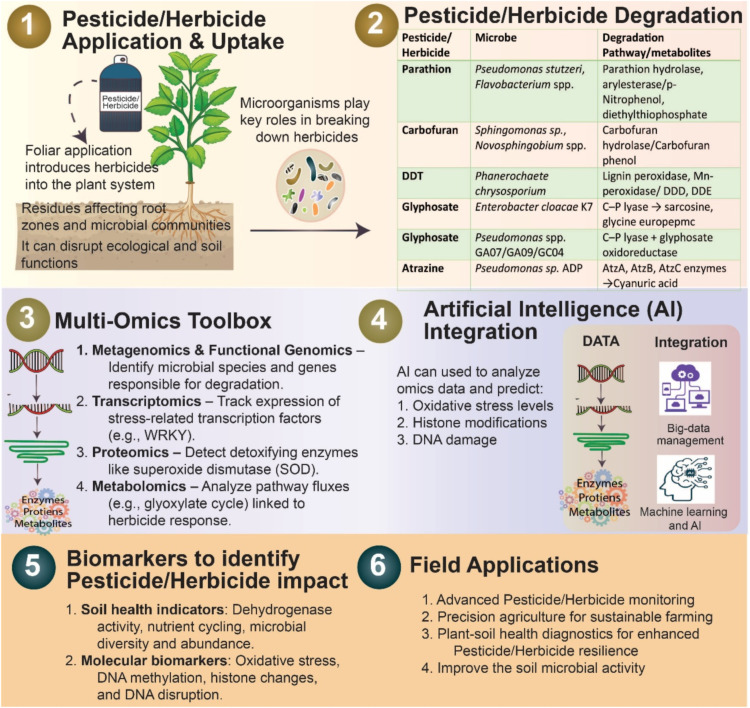
Systems-based approach to pesticides/herbicide degradation combining microbial bioremediation, omics-level biological diagnostics, and artificial intelligence.

Recent advances in biotechnology have facilitated the integration and interpretation of large datasets generated from diverse bioremediation studies across polluted environments. The application of omics-based tools and technologies, such as genomics, transcriptomics, proteomics, and metabolomics, provides an inclusive and systems-level understanding of microbial responses and adaptive mechanisms during contaminant degradation (Fig. 6). Each omics layer offers distinct insights: genomics identifies potential biodegradative genes; transcriptomics reveals gene expression patterns in response to pollutant stress; proteomics uncovers functional proteins involved in enzymatic pathways; and metabolomics deciphers the metabolic intermediates and end-products that reflect biochemical transformations.

Among these, systems biology a holistic and integrative framework has emerged as a powerful tool for interpreting complex interactions within microbial communities. By analysing multilayered omics data, systems biology enables predictive modelling and rational design of synthetic microbial consortia or bioaugmentation strategies tailored to specific pollutants and environmental conditions (Gangola et al., 2022). This approach enhances the efficacy, reliability, and scalability of bioremediation interventions. Moreover, systems biology supports the identification of keystone microbial taxa and core functional genes involved in pollutant detoxification, enabling researchers to monitor and manipulate key functional nodes within microbial networks. These insights are critical for the development of next-generation bioremediation strategies that harness indigenous microbial resources, naturally occurring enzymes, and environmental metabolites to restore ecosystem health sustainably.

### 6.1 Genomic-based approaches

Microbial degradation of pesticides is facilitated by specific genes or the enzymes, which are responsible for degrading these toxic compounds. Various bacteria, fungi, and actinomycetes possess distinct catabolic genes located on chromosomes, plasmids, or transposons, which enable them to metabolize pesticides effectively. The latest advancement in genomics enable the identification of novel genes responsible for pesticide degradation. The genomics study also provide the details description of genes, their metabolism and regulation pattern under the normal or stress conditions in both the cultivable and non-cultivable microbial species (Malla et al., 2022; Zhou et al., 2022; Hassan and Ganai, 2023). The native microbial communities adapt to pesticide-contaminated environments, developing enhanced degradation pathways through horizontal gene transfer (Anode and Onguso, 2021).

In the previous study authors reported about the pesticides degrading genes present in the microbial species. For example, Anas et al. (2024) reported about *opd* and *mcd* genes responsible for dichlorvos and carbofuran degradation found in *Serratia* sp. and *Pseudomonas* sp. respectively. Similarly, Jiang et al. (2019) reported about *ophB, ampA,* and *mpd*, which are responsible for the degradation of organophosphorus pesticides. Ridl et al. (2018) reported about the *bph* gene clusters present in *P. alcaliphila,* having potential to degrade biphenyl and its derivatives. In a study Zhu et al. (2019) reported about the genome analysis of strain *Pseudomonas* sp. KT 2440, which revealed the presence of multiple enzymes involved in the degradation of environmental contaminants.

Hao et al. (2018) reported the genome sequencing of *Lysinibacillus fusiform a* bacterial strain with deltamethrin-degradation potential and found that gene associated with sugar metabolism and exogenous chemical metabolism were highly enriched. Further in a study Liu et al. (2014) reported genome sequence of *Burkholderia* sp. which has the potential of methyl parathion degradation. Wang et al. (2019a) reported complete genome sequence of *Acinetobacter johnsonii* having cyprodinil pesticides degradation potential Shi et al. (2015) studied complete genome of pesticides degrading bacterial strain of *Pseudomonas stutzeri*. The genome study revealed different pesticides degrading gene such as methyl parathion hydrolase and organophosphorus hydrolase (*ophC2*,) which was responsible for organophosphorus pesticides degradation.

Metagenomics is an additional genomics-based method that facilitates the sequencing of cultivable or non-cultivable microbial samples (Liu et al., 2014; Shi et al., 2015). The metagenomic study helps in the exploration of biodegradation approach that remains hidden or unexplored and also play key role in the searching of core microbial taxa or the hub taxa of the pesticides contaminated biological samples (Mishra et al., 2021; Hassan and Ganai, 2023).

### 6.2 Transcriptomic-based approaches

Pesticides and other xenobiotic compounds in the surroundings significantly interfere with microbial populations and set off complex physiological and molecular processes. One of the main processes by which microorganisms develop to offset such pressures is transcriptional control, sometimes known as gene expression modulation. By regulating the expression of particular genes linked in the detection, transformation, degradation, and final mineralisation of harmful compounds, the adaptive response helps microorganisms to react dynamically to environmental pollutants (Hassan and Ganai, 2023).

Understanding the molecular mechanisms of stress responses, biodegradation, and detoxification requires detailed knowledge of how mRNA profiles are regulated in the presence of xenobiotics. High throughput transcriptomic analysis particularly RNA sequencing enables researchers to unrevealed the gene expression patterns, identify key genes involved in biodegradation pathways, and decode microbial adaptation mechanisms to pesticide contaminated environments. This knowledge not only enhances our understanding of microbial ecology but also guides successful bioremediation strategies (Cheng et al., 2018; Li et al., 2022; Hassan and Ganai, 2023).

In a global transcriptome analysis Cheng et al. (2018) investigated the gene expression dynamics of *Rhodococcus erythropolis* D310–1 during the degradation of chlorimuron-ethyl using RNA-Seq and qRT-PCR analyses. Their findings revealed that nearly 500 genes were upregulated throughout the degradation process. Notably, the study identified the expression of specific genes, including those encoding carboxylesterase, cytochrome P450, and glycosyltransferase, which are likely involved in the breakdown of chlorimuron-ethyl. Further, Li et al. (2022) accessed the transcriptomic response of *Pseudomonas nicosulfuronedens* in the presence herbicides of nicosulfuron. The RNA–Seq results showed up regulation of 1102 differentially expressed genes mainly belong to ABC transporter, sulphur metabolism and down regulation of 702 genes. Study conclude that the strain adopted acid metabolites production strategies during nicosulfuron degradation.

### 6.3 Proteomics-based approaches

Exposure to environmental stressors, including pesticide contamination, leads to significant variations in the protein expression profiles of microorganisms. Proteomic analyses enable researchers to characterize these changes and identify proteins synthesized in response to stress conditions. Examining such variations provides deeper insight into microbial stress responses, the effects of pesticide exposure on protein synthesis, and the regulatory proteins and genes involved in pesticide degradation pathways (Chandrasekhar et al., 2014; Bardot et al., 2015; Rodríguez et al., 2020; Castrejón-Godínez et al., 2022). Proteomic analysis has significantly advanced the identification of key enzymes involved in pesticide biodegradation, elucidated the primary metabolic pathways utilized for using pesticides as sources of carbon and energy, and highlighted proteins that play a role in mitigating cellular damage (Chandrasekhar et al., 2014; Rodríguez et al., 2020; Hassan and Ganai, 2023). In a study Castrejón-Godínez et al. (2022) evaluated the proteomic profile of Methyl parathion degrading *Burkholderia zhejiangensis* strains in the presence or absence of the pesticides. Protein expression patterns were analyzed using 2D-PAGE and MALDI-TOF. The proteomic analysis revealed differential expression of seventy-two proteins thirty-five in the absence of the pesticide and thirty-seven during methyl parathion exposure. These proteins were directly associated with the metabolic processes involved in pesticide degradation. Gangola et al. (2021) reported about Bacillus cereus, having degradation potential of cypermethrin, fipronil, imidacloprid and sulfosulfuron. During the study authors reported differential expression pattern of pesticides degrading proteins in the presence of cypermethrin.

### 6.4 Metabolomics in pesticide bioremediation

Metabolomics is the comprehensive qualitative and quantitative analysis of the metabolites ranging from small intermediates to end products within a biological system under specific physiological or environmental conditions (Rodríguez et al., 2020; Hassan and Ganai, 2023). These metabolite profiles are highly dynamic and responsive to external stimuli, such as environmental pollutants including pesticides exposure or biotic and abiotic stress conditions serving as direct indicators of cellular biochemical activity and linking genotypic variation with phenotypic expression (Wang et al., 2019a). Notably, the metabolomics investigates metabolic pathways composed of enzyme-catalysed biochemical reactions, where metabolites function as substrates, intermediates, or final products. Thus, analysing metabolite fluctuations provides insights into microbial and host metabolic responses and facilitates understanding of gene and protein functions in real biological contexts (Chen et al., 2015a). Previously, Feng et al. (2017) reported the significance of metabolomics in the degradation of pesticides Nicosulfuron using the fungal strain *Penicillium oxalicum* and confirmed that acidolysis of nicosulfuron was led by oxalate secretion. Chen et al. (2015a) reported a new approach of Cyhalothrin degradation using the strain of *Bacillus thuringiensis* strain after metabolomics study.

Although omics-based technologies such as genomics, metagenomics, proteomics, and metabolomics have significantly advanced our understanding of microbial mechanisms involved in pesticide degradation. However, several limitations still constrain their broader application. One of the primary challenges is the high cost of high-throughput sequencing platforms and computational resources, generation of large-scale omics data and their processing (Li et al., 2025; Smith et al., 2025). The analysis of big data generated during metagenomics, metabolomics and proteomics requires expertise in system biology, informatics and molecular biology for the better understanding (Li et al., 2025). The extraction of high-quality DNA, RNA or nucleic acids from the pesticides contaminated sites are one of the challenging tasks, which not only affect the amplification process but also hampers downstream analyses (Alidoosti et al., 2024). Moreover, reproducibility and comparative interpretation of data generated during fluctuating environmental condition is another hurdle, because these variation influences the expression pattern of genes and microbial communities’ structure (Soufi et al., 2024; Ramond et al., 2025).

Despite these challenges the latest advancement in omics technologies, high sequence accuracy, development of informatics pipeline and cost reduction strategies are gradually enhancing the reliability and applicability of omics tools in understanding and optimizing microbial pesticide degradation processes.

## 7 Application of gene editing technologies in pesticide remediation

The modification of target genes in the suitable microorganisms either through insertion or deletion by the use of gene editing strategies has become an emerging approach in the microbial based bioremediation of chemical pesticides. The integration of advanced omics technologies further facilitates the identification of microorganisms, gene, and enzymatic pathways responsible for the pesticide’s degradation (Li et al. 2022; Wu et al. 2023; Hassan and Ganai 2023). The gene editing technologies such as CRISPR-Cas, ZFN (zinc finger nucleases), and TALEN (transcription activator-like effector nucleases) are most commonly used for the genetic modification (Broeders et al. 2020). CRISPR/Cas9 technology enables insertion, deletion, or activation of genes responsible for pesticides degradation. Additionally help in the up-regulation of pesticides degradation pathways by inserting strong promoters or knocking out the repressor genes (Wong, 2018). Gene editing technique utilizes custom-designed guide sequences that are complementary to the target gene, enabling site-specific modifications through mechanisms like homologous recombination, which allow for the insertion or deletion of specific DNA segments (Bier et al., 2018).

The CRISPR-Cas system is widely recognized as the most effective and extensively used gene-editing technology (Wei et al., 2022; Hassan and Ganai, 2023). It is generally classified into three main types Type I, Type II, and Type III each containing multiple subtypes that exhibit unique functional characteristics (Khan et al., 2022). The specificity of each system is determined by the unique Cas (CRISPR-associated) protein it employs (Cooper et al., 2018). Among these, the Cas9 protein, an RNA-guided DNA endonuclease, plays a pivotal role by recognizing and cleaving foreign DNA sequences. This process is facilitated by a small RNA molecule known as CRISPR RNA (crRNA), which is derived from a 30–40 base pair direct repeat sequence. The crRNA guides Cas9 to its complementary target site, enabling precise DNA interference or cleavage (Zhang et al., 2018). In contrast, other gene-editing technologies such as Zinc Finger Nucleases (ZFNs) and Transcription Activator-Like Effector Nucleases (TALENs) employed engineered restriction enzymes to create double-strand breaks at precise locations within the DNA sequence (Banerjee et al., 2018). These gene-editing technologies aim to enhance microbial functions by introducing a broader range of genetic traits, thereby expanding their metabolic capabilities and environmental adaptability (Basu et al., 2018).

In the previous studies authors uses gene editing technology to improve efficacy of pesticides degradation. For example, in a study, Zhang et al. (2023) successfully utilized CRISPR/Cas9 system to knock out the *opd B* gene, crucial for the degradation of organophosphorus insecticides. This genetic modification resulted in a mutant strain with over 30 % efficiency in homologous recombination, demonstrating the potential for enhanced biodegradation capabilities. Fu et al. (2022) used CRISPR-Cas genome editing to developed enzymatic cascade-based assays for organophosphorus pesticides, enabling the investigation of degradation mechanisms for compounds such as paraoxon, demeton, and dichlorvos (Fu et al., 2022). In a study ZFNs techniques had been used to modify the *opdH* gene in *Arthrobacter* sp. HM01 to increased chlorpyrifos degradation (Mali et al., 2022). Similarly various authors reported the significance of gene editing technology in improving pesticides degradation.

Till date, numbers of genetically engineered microorganisms have been employed for the remediation of contaminated sites, nevertheless, the plausible hazards of genetic material exchange have limited the successfulness at field scale (Singh et al., 2011). The introduction of genetically modified microorganisms could have undesirable consequences on natural environment and human health (Lerner et al., 2024). The release of engineered microorganisms under certain conditions needed for the purpose of bioremediation may favor the selection of antibiotic and other biocide resistant microorganisms (Davison 2005). One of the most commonly expected risks of engineered bacteria is the horizontal transfer of genes concerned with the resistance to pesticides, heavy metals, and antibiotics to other naturally existing non-target bacteria through the process of conjugation, transformation, and transduction, aggravating environmental concerns (Wu et al., 2021; Carolak et al., 2025). The unintended release of engineered microbes may modulate the structure and composition of native microbial population, hence the disruption of associated key biological processes (Chemla et al., 2025). The uncontrolled spread of engineered traits could lead to negative environmental consequences, especially in wastewater treatment plants, affecting downstream aquatic environment (Thakur et al., 2025).

The engineered microbes tailored specifically for a given contaminant or habitat may behave unexpectedly after the introduction into the natural environmental condition, compete with naturally dwelling microorganisms, and modulate nutrient cycling. The other facet of risk is concerned with the biosynthesis of some hazardous intermediate compounds during the degradation of a synthetic chemicals such as per- and polyfluoroalkyl substances (PFAS), posing undesirable effects on human health and environment (Tushar et al., 2024). Further, considering poor detectability in short time span and unavailability of effective methods warranted for better prediction, the employment of genetically engineered microorganisms for the bioremediation is still controversial (Liu et al., 2019). The employment of engineered microorganisms for the purpose of environmental remediation is subject to strict regulatory legislations designed for ensuring better human health and environment (Saxena et al., 2019; Hoffmann et al., 2023). Although the frameworks differ country wise, the basic steps involves exhaustive examination, appraisal of the plausible risks prior to field scale translation, mitigation of industrial accidents, and bio-safety assessment such as effect on environment and genetic stability (Sayler and Ripp, 2000). Regulatory bodies such as U.S. Environmental Protection Agency (EPA) and other organizations globally mandate extensive investigations on the survival ability, genetic integrity, and environmental consequences of engineered microorganisms (Wu et al., 2021 ). The attitude of the public regarding the safety of engineered microbes can considerably influence the decision and delay the implementation. Therefore, intensive risk-benefit assessment along with the important steps needed for the formulation of transparent guidelines should be practiced for the judicious application of engineered microorganisms.

## 8 Significance of artificial intelligence (AI) in pesticides bioremediation

AI is revolutionizing the field of pesticides bioremediation by enhancing detection, degradation, and management processes. AI technologies, including machine learning (ML), artificial neural networks (ANN), deep learning are being integrated with bioremediation techniques to optimize the degradation of pesticide-contaminated environments. These advancements are crucial for addressing the environmental and health risks posed by pesticide residues (Banerjee et al., 2024). AI is playing a crucial role in enhancing efficacy of microbial based pesticides degradation.

AI play significant role in selection of microbial strains, microbial genes or optimizing the microbial processes by analysing the huge data set of metagenomics, transcriptomics or metabolomics and other technologies (D’Urso and Broccolo, 2024; Yu et al., 2025). Additionally, AI take part in predicting degradation pathways involved in pesticide degradation, which help in understanding the enzymatic processes and optimize them for better efficiency (Kumar and Sachan, 2021). The AI models helps in predicting the formation of non-toxic byproducts, ensuring complete mineralization of pesticides into harmless substances (Kumar and Sachan, 2021).

As for the effective pesticides degradation, the growth and tolerance of microorganism is an essential requirement, AI can optimize biotic and abiotic factors such as temperature, humidity, and pH to enhance microbial activity and degradation rates (Sehrawat et al., 2021). AI improves microbial bioremediation of pesticide degradation through real-time monitoring and optimization of environmental parameters. It persistently gathers and evaluates data on microbial activity, pollutant levels, and environmental variables, facilitating modifications in pH, temperature, and nutrient concentrations to optimize microbial efficacy. Moreover, predictive modelling anticipates remediation results, enabling proactive modifications to enhance the degradation process. The incorporation of AI diminishes operational expenses and enhances the accuracy and sustainability of bioremediation initiatives for pesticide contamination (Akintola, 2024). As it is well reviewed that pesticides exposure to the food chains leads to various health concern and the accumulation of pesticides leads to contamination or eutrophication in the water ecosystem. Therefore, early detection of pesticides exposure could be a crucial step for pesticides management. Now a days AI has been frequently utilized for the early detection of pesticides by the use of various ML algorithms in very short time in compared to the traditional approaches (Lapcharoensuk et al., 2023).

Machine Learning (ML), a rapidly advancing branch of AI, enables systems to learn and improve over time through trial and error. By analyzing past experiences and leveraging accumulated data, ML significantly enhances the decision-making and operational efficiency of AI-driven systems (Greener et al., 2022). Consequently, the incorporation of machine learning is vital for the precise operation of AI across multiple domains, including the identification of environmental pollutants. For example, Lapcharoensuk et al. (2023) uses of portable near-infrared (NIR) spectrometers coupled with ML algorithms for the on-site, detection of pesticide residue. Sahin et al. (2022) used surface-enhanced Raman spectroscopy (SERS) or colorimetric arrays analysed by ML, for the rapid or accurate detection of pesticides. The application of Machine Learning (ML) models offers significant advantages, particularly in their ability to analyse complex and nonlinear relationships between pesticide properties and their detectability relationships often overlooked by conventional risk assessment methods. For example, an Artificial Neural Network (ANN) model demonstrated remarkable accuracy, achieving a 99.94 % correlation with experimental data in predicting the degradation of chlorpyrifos. The ML algorithms have the capability to access or interpret the dataset of various origins such as spectral, chemical and physical nature. For example, Spectral analysis as it is a comprehensive AI methodology that quantifies the wavelengths of light absorbed or reflected by a substance, such as a pesticide or heavy metal. This approach enables more efficient identification and quantification of specific chemical compounds across various sample types and is typically faster than traditional methods like liquid chromatography–mass spectrometry (LC-MS) and gas chromatography–mass spectrometry (GC–MS) (Hassani et al., 2017).

Deep learning (DL) has emerged as a transformative tool in pesticide residues analysis by leveraging advanced algorithms. In recent past, researchers have developed models that improve the efficiency and accuracy of pesticide degradation predictions, as well as the identification of pesticide residues in various environments (Ye et al., 2022). For example, Zhou et al. (2023) enabled the miniature mass spectrometer that hep in the rapid analysis of pesticides present on the vegetable surface. Similarly, Houngkamhang and Phasukkit (2022) reported about the portable deep learning-driven ion-sensitive field-effect transistor that will help in the quantification of carbaryl pesticides.

The integration of Artificial intelligence and their subset such as ML and DL enhance the predictive accuracy which can led to reduction in operational cost and promote sustainable pesticides degradation. AI based predictions is highly dependent upon the data quality, validation, training and standardization of data. Although most of the available datasets remain fragmented and primarily based on laboratory conditions and lack field-scale validation (Greener et al., 2022; Banerjee et al., 2024). Therefore, to fully harness the potential of AI the future research must be emphasized on the high-quality data, model validation, and integration of AI predictions with experimental and field-based studies that will help in efficient and sustainable pesticides bioremediation.

## 9 Conclusion and future prospective

Microorganism-based bioremediation presents a sustainable and eco-friendly alternative to conventional methods for mitigating pesticide contamination. However, its large-scale implementation faces challenges due to inconsistent performance, ecological uncertainty, and regulatory constraints. Environmental variability, such as fluctuations in temperature, pH, moisture, and soil organic matter, often inhibits the survival, colonization, and functional efficiency of introduced microbial strains. Additionally, competition with indigenous microorganisms can suppress the activity of bioremediating microbes, especially in complex soil environments. To overcome these limitations, synthetic or naturally derived microbial consortia are being developed to harness the synergistic metabolic interactions among diverse species. These consortia enable co-metabolism of multiple pesticide compounds and provide resilience under variable environmental conditions. Therefore, the selection of suitable microorganisms, compatibility of different microorganism in the consortia or the selection of core microbiome is required for brief investigation.

Genetically edited microorganisms offer significant potential for advancing bioremediation, as they can be engineered for enhanced pollutant degradation, improved metabolic efficiency, and better adaptation to harsh environmental conditions. However, despite these promising advantages, their release into natural ecosystems raises notable concerns regarding potential ecological risks. These include unintended horizontal gene transfer, disruption of native microbial communities, alteration of ecological balance, and the persistence of modified strains in non-target environments. Therefore, rigorous biosafety assessments, containment strategies, and regulatory oversight are essential prior to field application to ensure that genetically edited strains do not introduce unforeseen ecological disturbances. Future research should thus prioritize not only improving degradation efficiency but also elucidating and mitigating ecological risks to support the safe and sustainable application of gene-edited microorganisms in bioremediation.

Advances in latest omics technologies such as genomics, transcriptomics, proteomics, and metabolomics enhance the understanding about microbial communities’ structure, identifying the genes and pathways of pesticides degradation. However, their practical implementation is constrained by high operational costs, the generation of massive datasets, complex data interpretation requirements, and difficulties in integrating multi-omics information under variable environmental conditions. To address these challenges is urgent need to develop standard protocols, generation of cost-effective protocols and programming of advanced bioinformatics pipelines, which can interpret large-scale, heterogeneous environmental data.

Microbial bioremediation, supported by omics and AI-driven approaches, offers a promising path toward sustainable pesticide management. AI and ML tools are also being employed to predict microbial behaviour, model degradation pathways, and design of optimal consortia based on environmental parameters and contaminant profiles. These computational technologies significantly accelerate the identification of high-performance strains and support precision-based, site-specific bioremediation strategies. Nevertheless, these computational models rely heavily on data quality, validation accuracy, and representativeness limitations that may result in biased predictions or oversimplified outcomes if not critically addressed.

Future research should emphasize the integration of big data analytics, biosensor-based monitoring, and systems biology framework to enhance the predictability, efficiency, and field applicability of microbial consortia. There is also a critical need to improve machine learning training datasets to accurately quantify pesticide concentrations, classify pesticide types, and provide decision-support solutions for economically viable and sustainable pesticide management.

## Funding sources

No external funding received for this study.

## CRediT authorship contribution statement

**Ajay Kumar:** Conceptualization, Supervision, Validation, Writing – original draft, Writing – review & editing. **Manoj Kumar Solanki:** Conceptualization, Writing – original draft, Writing – review & editing. **Manish Kumar:** Investigation, Writing – original draft, Writing – review & editing. **Amit Kaushik:** Data curation, Formal analysis. **Aditi Arya:** Data curation, Formal analysis. **Mahaswetta Saikia:** Data curation, Formal analysis, Writing – review & editing. **Vivek Kumar Gaur:** Investigation. **Rahul Prashad Singh:** Data curation, Formal analysis, Writing – review & editing. **Sandeep Kumar Singh:** Data curation, Formal analysis, Writing – review & editing. **Vipin Kumar Singh:** Conceptualization, Writing – original draft, Writing – review & editing. **Laurent Dufossé:** Funding acquisition, Supervision, Validation.

## Declaration of competing interest

The authors declare that they have no known competing financial interests or personal relationships that could have appeared to influence the work reported in this paper.
